# Polysomy of chromosome 17 in breast cancer tumors showing an overexpression of ERBB2: a study of 175 cases using fluorescence *in situ *hybridization and immunohistochemistry

**DOI:** 10.1186/bcr996

**Published:** 2005-01-26

**Authors:** Marta Salido, Ignasi Tusquets, Josep M Corominas, Marta Suarez, Blanca Espinet, Cristina Corzo, Meritxell Bellet, Xavier Fabregat, Sergi Serrano, Francesc Solé

**Affiliations:** 1 Laboratori de Citogenètica i Biologia Molecular, Servei de Patologia, Hospital del Mar, IMAS, Barcelona, Spain; 2 Escola de Citologia Hematològica S Woessner-IMAS, Hospital del Mar, IMAS-IMIM, Barcelona, Spain; 3 Unitat de Recerca translacional en tumors sòlids-IMAS, Barcelona, Spain; 4 Servei d'Oncologia Mèdica, Hospital del Mar, IMAS, Barcelona, Spain; 5 Universitat Autònoma de Barcelona, Barcelona, Spain; 6 Servei de Patologia, Hospital del Mar, IMAS, Barcelona, Spain

**Keywords:** breast cancer, ERBB2 gene, fluorescence *in situ *hybridization, immunohistochemistry, polysomy 17

## Abstract

**Introduction:**

One of the most common genetic aberrations associated with breast cancer is the amplification and overexpression of the ERBB2 proto-oncogene located at chromosome 17, bands q12-21. The amplification/overexpression occurs in 25 to 30% of all breast cancers. In breast cancer, aneusomy of chromosome 17, either monosomy or polysomy, is frequently observed by conventional cytogenetics and fluorescence *in situ *hybridization (FISH). The aim of this study was to discover whether or not numerical aberrations on chromosome 17 have a correlation to the amplification or overexpression of the ERBB2 gene and to analyze their clinical implications in subgroups showing 2+ or 3+ positive scores by immunohistochemistry (IHC).

**Methods:**

We used FISH on a series of 175 formalin-fixed paraffin-embedded breast carcinomas to detect ERBB2 amplification, using a dual-probe system for the simultaneous enumeration of the ERBB2 gene and the centromeric region of chromosome 17, as well as using IHC to detect overexpression. We analyzed clinical and pathological variables in a subgroup of patients with 2+ and 3+ IHC scores (147 patients), to describe any differences in clinicopathological characteristics between polysomic and non-polysomic cases with the use of the χ^2 ^test.

**Results:**

We found 13% of cases presenting polysomy, and three cases presented monosomy 17 (2%). According to the status of the ERBB2 gene, instances of polysomy 17 were more frequently observed in non-amplified cases than in FISH-amplified cases, suggesting that the mechanism for ERBB2 amplification is independent of polysomy 17. Polysomy 17 was detected in patients with 2+ and 3+ IHC scores. We found that nodal involvement was more frequent in polysomic than in non-polysomic cases (*P *= 0.046).

**Conclusions:**

The determination of the copy number of chromosome 17 should be incorporated into the assesment of ERBB2 status. It might also be helpful to differentiate a subgroup of breast cancer patients with polysomy of chromosome 17 and overexpression of ERBB2 protein that probably have genetic and clinical differences.

## Introduction

Proto-oncogenes and tumor suppressor genes are two classes of genes with central roles in the regulation of cell growth. One of the most common genetic alterations associated with human breast cancer is the amplification of the ERBB2 proto-oncogene [1]. The ERBB2 gene located on 17q12-q21 encodes a 185 kDa transmembrane tyrosine kinase receptor [2,3]. This protein is a member of the epidermal growth factor receptor family [4] that comprises four homologous receptors: HER1 (ERBB1), ERBB2 (ERBB2), HER3 and HER4. These receptors are involved in the activation of complex signaling pathways, essential for cell survival and for the regulation of normal breast growth and development [5-7]. Several studies performed in various laboratories have demonstrated that 25 to 30% of all breast and ovarian malignancies show the amplification and overexpression of this gene [8,9]. Amplification of the ERBB2 gene is found in more than 90% of cases that have ERBB2 protein overexpression [10,11], but in normal breast the expression of ERBB2 is due to a transcriptional activation [12]. ERBB2 overexpression in women with both node-positive [8,9] and node-negative [13] breast cancer is associated with a poor prognosis, and several studies have found a correlation between ERBB2 overexpression and a shorter disease-free period and shorter overall survival [14,15].

ERBB2 overexpression and/or gene amplification is an indication for trastuzumab (Herceptin; Genentech, South San Francisco, CA, USA) therapy in patients with metastatic breast cancer [16,17]. Clinical trials combining trastuzumab and chemotherapy have been initiated, based on preclinical data about potentially enhanced anti-tumor activity when anti-ERBB2 antibodies were combined with chemotherapeutic agents [18-20].

There are different methods available to evaluate ERBB2 status [21], although immunohistochemistry (IHC; for protein overexpression) and fluorescence *in situ *hybridization (FISH; for gene amplification) offer several advantages, because the aberration can be evaluated directly in malignant cells taken from archival breast cancer specimens. Reports of false-positive Herceptest cases led to suggestions that Herceptests yielding a 2+ score should also be studied by FISH [22]. Tubbs and colleagues [23], called for the US Food and Drug Administration (FDA) to mandate the retraction of the earlier accepted criteria for trastuzumab therapy, namely that of Herceptests yielding a 2+ score, unless those cases were also confirmed by FISH.

The FDA-approved FISH assay, PathVysion (Vysis, Inc., Downers Grove, IL, USA), is a dual-probe system for the simultaneous enumeration of the ERBB2 gene and the centromeric region of chromosome 17, defining ERBB2 amplification as a ratio of ERBB2 gene copies per chromosome 17 centromere [24]. Another FDA-approved FISH assay, INFORM (Ventana Medical systems, Tucson, AZ, USA), defines ERBB2 amplification as a mean absolute ERBB2 gene copy number of more than four spots per nucleus, without centromere 17 correction. FISH with the PathVysion probe and immunohistochemical assay with Herceptest are highly concordant for cases showing 3+ IHC scores and for negative cases, although the 2+ IHC score group includes both ERBB2 amplified and non-amplified tumors, showing a relatively high rate of discordance [25,26]. The mechanisms for ERBB2 expression in non-amplified tumors scored 2+ by IHC are unclear and may involve increased gene dosage by chromosome 17 polysomy [27].

In breast carcinoma, chromosomal aneusomy – either monosomy or polysomy – is frequently observed by conventional cytogenetics and FISH [28,29]. Sauer and colleagues [30,31], found that an abnormal number of copies of chromosome 17 have a low impact on ERBB2 gene and its expression, but more studies are necessary to confirm these results.

The aim of our study was to analyze the role of polysomy 17 in ERBB2 protein expression and its implication in ERBB2 gene status in a series of patients with tumors scoring 2+/3+ by IHC.

## Materials and methods

### Patients' characteristics

This is a prospective analysis of 175 breast cancer patients, consecutively treated at the Hospital del Mar of Barcelona between August 2000 and August 2003.

### Specimens

We studied specimens taken from 175 prospective cases of human breast cancer. Overexpression was determined by IHC, and amplification was studied by FISH.

The breast cancer specimens used in this study were fixed with 4% buffered formalin, and the tissue was then embedded in paraffin. Sections 4 to 6 μm thick were cut from the tissue, mounted on silanized slides and then deparaffinized in a xylene series, followed by immersion in 100% ethanol. Then we serially sectioned a hematoxylin/eosin (H&E)-stained tissue section, an IHC tissue section and a FISH tissue section from each of the patient samples.

### IHC assay

This method involved the application of primary ERBB2 antibody (rabbit anti-human ERBB2 oncoprotein; DakoCytomation, Glostrup, Denmark) at 1:200 dilution. This antibody was evaluated with the dextran/peroxidase technique (Dako Envision). We used negative and positive controls for ERBB2 overexpression in all samples. The IHC technique was applied with an automated system (Tech Mate 500) after antigen retrieval at 110°C for 1 min in a wet autoclave. When FDA approved the Herceptest kit (DakoCytomation) as a diagnostic method to detect ERBB2 overexpression, we began to use it. As a single institution, we observed an excellent correlation between these two IHC detection systems.

Membrane staining was interpreted as ERBB2 oncoprotein expression. To evaluate the immunostaining for ERBB2 antibody, we considered the intensity and type of membrane expression. Expression was recorded as follows: score 0, negative staining; score 1, local positivity and incomplete membrane staining; score 2, moderate, complete membrane staining; score 3, strong, complete membrane staining.

### FISH assay

The Pathvysion probe was used. This probe consists of two different probes, one with the centromeric α-satellite probe, specific for chromosome 17 (Spectrum green), and a locus-specific probe from the ERBB2 gene (Spectrum orange). The probe was provided denatured, in single-stranded DNA.

Deparaffinized tissue sections were treated with 0.2 M HCl and then with sodium thiocyanate, to eliminate salt precipitates. Pretreated slides were incubated for 10 min in a solution of proteinase K at 37°C. The slides were then postfixed in buffered formalin.

Pretreated tissue sections and probes were denatured at 78°C for 5 min and hybridized overnight at 37°C on a hotplate (Hybrite chamber; Vysis). Washes were performed for 2 min at 72°C in a solution of 2 × SSC/0.3% Nonidet P40. Tissue sections were counterstained with 10 μl of 4,6-diamino-2-phenylindole (DAPI counterstain; Vysis).

Results were analyzed in a fluorescent microscope (Olympus BX51) with the Cytovysion software (Applied Imaging, Santa Clara, CA, USA). Tissue sections were scanned at low magnification (×100) with DAPI excitation to localize those areas where histopathological characteristics had been established by examining a serially sectioned H&E-stained tissue section from the same patient.

ERBB2 amplification was calculated by a ratio dividing the most frequent value for ERBB2 spots per nucleus by the most frequent value of chromosome 17 centromere spots per nucleus. A minimum of 60 nuclei were scored. Amplification of ERBB2 gene was considered when the ratio was 2 or more, in accordance with the manufacturer's recommended scoring system. We considered polysomy 17 when the cells had three or more copy numbers of centromeres for chromosome 17 per cell.

### Statistical analysis

A total of 147 patients with 2+ and 3+ IHC scores out of 175 of the complete cohort were analyzed, ensuring that all the polysomies of chromosome 17 were detected in this subgroup of patients. Table 1 describes the following clinicopathological parameters: clinical stage, nodal status, histological grade, estrogen receptor (ER), progesterone receptor (PgR), p53 protein and relapse of the disease, and age description. We performed a concordance analysis using the χ^2 ^test between prognostic variables and the presence or absence of polysomy 17 to detect any differences between polysomic and non-polysomic subgroups.

## Results

To validate the FISH technique, in a first screening step we performed FISH on 50 specimens that represented all of the immunohistochemical subgroups (0, 1+, 2+ and 3+). For subsequent specimens we performed FISH on 2+ and 3+ IHC subgroups. The exclusion of the subgroups with scores of 0 and 1+ was based on our previous finding that none of those cases had ERBB2 gene amplification [25]. This selection process resulted in the under-representation of IHC-negative and 1+ specimens for the FISH assay and a high proportion of 2+/3+ cases.

**Figure 1:**
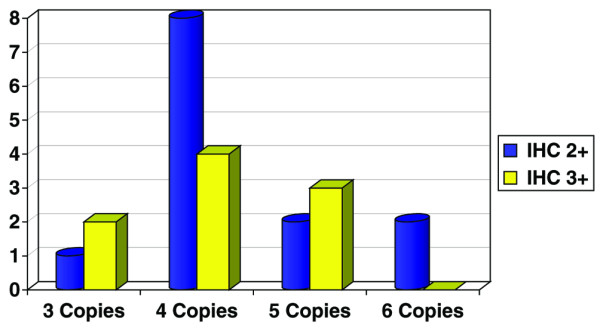
Distribution pattern of polysomy 17 in our series of 147 patients studied by fluorescence *in situ *hybridization.

Among the 175 specimens studied by IHC and FISH, 22 cases showed polysomy of chromosome 17 (13%), and three cases presented monosomy (2%) (Table 2). Of the 175 cases, 147 showed an IHC score of 2+ or 3+. The distribution of chromosome 17 copy numbers in this 2+/3+ subgroup is illustrated in Fig. 1. Most of the cases with polysomy 17 had four copies of chromosome 17 per cell.

We observed that all cases with polysomy of chromosome 17 had an IHC score of 2+ or 3+. Thirteen of 22 cases with polysomy revealed an IHC score of 2+, and the remaining 9 cases had an IHC score of 3+. In a subgroup of 78 patients having IHC scores of 2+, 13 of 78 (17%) showed polysomy of chromosome 17 with increased ERBB2 gene copies, but without gene amplification. In a subgroup having IHC scores of 3+, 9 of 69 cases (15%) presented polysomy 17. In patients with monosomy of chromosome 17, two cases presented IHC scores of 2+, and one presented an IHC score of 3+, without ERBB2 gene amplification.

According to ERBB2 gene status, all cases with polysomy and 2+ IHC scores were considered to be normal for ERBB2 gene amplification (the ratio was 2 or less). Of the cases considered normal by FISH, 15% presented polysomy 17, and 10% of the amplified cases showed amplification and polysomy 17 simultaneously (Table 2). Within the subgroup having scores of 3+ and polysomy, only one case presented non-amplification of the ERBB2 gene.

We compared the polysomic subgroup with the non-polysomic subgroup, to determine whether there were differences in the clinicopathological features. After clinical analysis of the 147 patients who were 2+/3+ by IHC, we found that nodal involvement was significantly associated with polysomy 17 (*P *= 0.046). Furthermore, patients with polysomy 17 also showed a non-statistical trend toward relapse (*P *= 0.181). No statistically significant correlation was observed between histological grade, ER, PgR and p53 variables and the polysomy of chromosome 17 (Table 3).

## Discussion

In our study, 175 cases diagnosed as invasive breast cancer were examined to determine the frequency of chromosome 17 polysomy in different ERBB2 IHC subgroups. In our series we found 22 specimens with polysomy 17 (13%) and three cases with monosomy 17 (2%). In a series from Wang and colleagues [31], aneusomy was very common (more than 50%), but only 10 of 189 cases (5%) showed high polysomy (at least 3.76 signals of centromere 17 per cell). Series from Watters and colleagues [32] presented a high proportion of aneusomy 17 (54%); most of those presented polysomy, but the highest proportion fell into the 2.00 to 3.00 chromosome 17 copy numbers category. In those two series [31,32], they considered polysomy with a mean chromosome copy number near to disomy. The reason for the discordance with our series (13% versus 50%) could be explained by the consideration of polysomy 17 when the cells had three or more copy numbers of centromeres for chromosome 17 per cell, in agreement with other authors [22,26,27,29].

Cases with polysomy 17 could be considered to be amplified at a low level by absolute criteria, but they had to be classified as non-amplified when the number of ERBB2 copies was corrected for the number of chromosome 17 centromeres. FISH, using the dual probe for the ERBB2 gene and the centromere of chromosome 17, offers the greatest resolution in detecting alterations of the ERBB2 gene in breast tumors because it allows true amplification to be differentiated from polysomy 17. The FISH method is less affected by tissue variables than the IHC method, and it has emerged as the gold standard for the assessment of ERBB2 status in breast cancer.

We observed polysomy 17 in non-amplified cases more frequently than in FISH amplified cases (15% versus 10%), suggesting that the mechanism for amplification of the ERBB2 gene is independent of polysomy.

In our series, polysomy 17 was frequently associated with a 2+ and 3+ IHC score. We had 13 of 22 polysomic cases that showed IHC 2+ scores with FISH-negative results. In contrast, other authors [33,34] found that the incidence of polysomy 17 in 2+ non-amplified cases was similar to the incidence of polysomy 17 in IHC-negative cases, suggesting that weak overexpression (2+) without gene amplification is not secondary to chromosome 17 polysomy. Our findings support the concept that polysomy 17 is a genetic aberration independent of IHC status but that it might have a role in ERBB2 expression in weakly positive (2+) and in strongly positive (3+) cases. It is probable that there are non-amplified cases analyzed by FISH with false positive IHC (2+) scores as a result of polysomy 17. Polysomy 17, in the absence of ERBB2 amplification, would result in an increase in protein production to such level that it might be IHC stained as a positive result (2+).

We identified only one such case, IHC scored at 3+, non-amplified by FISH, and showing polysomy 17. It is reasonable that the copy number of chromosome 17 was associated with an increased level of ERBB2 protein but not with ERBB2 gene amplification. With regard to this case, we are in agreement with Lal and colleagues [33] and Varshney and colleagues [34], who suggested that the findings of strongly positive IHC staining (3+) without gene amplification might be due, in part, to the increased copy numbers of chromosome 17, resulting in ERBB2 overexpression.

In our study of 147 patients having tumors with 2+/3+ IHC scores, we found a statistical association between polysomy 17 and nodal involvement, and we observed that this subgroup of patients had a trend towards relapse more frequently than the non-polysomic cases. Only a few studies have focused on the analysis of the prognostic value of chromosome 17 aneusomy, and specifically on polysomy 17. In a large series from Watters and colleagues [32], abnormalities in chromosome 17 copy numbers were associated with grade III carcinomas and ER negativity, but they were without significant impact on survival. Other studies proposed that aneusomy of chromosome 17 is associated with poor prognostic factors in breast cancer [30].

## Conclusions

Breast tumors scoring at 2+ or 3+ by IHC should be analyzed by FISH for ERBB2, preferably with a dual-probe system capable of detecting aberrations in chromosome 17 that could affect ERBB2 expression. The accurate measurement of chromosome 17 polysomy might also be helpful in determining subgroups of patients with genetic and clinical differences, which would permit their inclusion in future clinical trials.

## Abbreviations

ER = estrogen receptor; FDA = US Food and Drug Administration; FISH = fluorescence *in situ *hybridization; H&E = hematoxylin/eosin; IHC = immunohistochemistry; PgR = progesterone receptor.

## Competing interests

The author(s) declare that they have no competing interests.

## Authors' contributions

MS conceived and designed the study, performed FISH analysis, interpretated the data and drafted the article. IT coordinated the study and was significantly involved in patient recruitment and the correlation of clinical data with experimental findings, also taking a role in supervising and final approval of the article. JMC contributed to the design of the study, analysed the immunohistochemistry experiments providing several data presented in the publication, and took a role in supervising and final approval of the article. MS contributed to the analysis of the data and was involved in patient recruitment. CC contributed to analysis and interpretation of the data and revised the article. BE contributed to interpretation of the data and revised the article. MB contributed to the recruitment of patients and revised the paper. XF is the head of the Oncology Department of Hospital del Mar de Barcelona. FS revised the paper, giving final approval of the version to be submitted, and is the head of the Cytogenetics Laboratory of Hospital del Mar de Barcelona. SS is the head of the Pathology Department of Hospital del Mar de Barcelona. All authors read and approved the final manuscript.

## Figures and Tables

**Table 1:** Correlation of chromosomal aneusomy with clinicopathological parameters

Parameter	Total	Disomy	Polysomy	Monosomy
Stage				
I	39	36	3	0
II	46	35	11	0
III	35	28	5	2
IV	7	6	0	1
Nodal status				
pN-	35	29	5	1
pN+	113	99	12	2
HG				
I	16	15	1	0
II	55	42	12	1
III	47	39	6	2
ER				
-	60	50	9	1
+	85	71	12	2
PgR				
-	87	72	12	3
+	58	48	10	0
p53				
-	87	72	14	1
+	56	8	46	2
Relapse				
No	110	94	14	2
Yes	36	27	8	1

The mean age of patients was 62 years (range 30 to 94). ER, estrogen receptor; HG, histological grade; PgR, progesterone receptor.

**Table 2:** Polysomy 17 according to immunohistochemical scoring and fluorescence *in situ *hybridization analysis

	Polysomy 17			Non-polysomy 17	
IHC score	FISH normal	FISH amplified	Polysomy 17 (%)	FISH normal	FISH amplified

0	0/8	0/0	0	8/8	0/0
1+	0/20	0/0	0	20/20	0/0
2+	13/66	0/12	17	53/66	12/12
3+	1/1	8/68	15	0/1	60/68
Total	14/95 (15%)	8/80 (10%)	13	81/95 (85%)	72/80 (90%)

FISH, fluorescence *in situ *hybridization; IHC, immunohistochemistry.

**Table 3:** Concordance analysis using the χ^2 ^test between the presence and absence of polysomy 17 and clinicopathological variables in a subgroup of 2+/3+ IHC patients

Parameter	Non-polysomy (%)	Polysomy (%)	*P*
HG			
I	15.6	5.3	
II	43.8	63.2	0.243
III	40.6	31.6	
Nodal status			
pN-	61.3	35.3	0.046
pN+	38.7	64.7	
ER			
-	41.7	40.9	0.947
+	58.3	59.1	
PgR			
-	60	54.5	0.632
+	40	45.5	
p53			
-	61	63.6	0.817
+	39	36.4	
Relapse			
No	77	63.6	0.181
Yes	23	36.4	

ER, estrogen receptor; HG, histological grade; PgR, progesterone receptor.
